# Scrotal Abscess Following Laparoscopic Appendectomy in an Older Adult Without Perforated Appendicitis: A Case Report

**DOI:** 10.7759/cureus.102848

**Published:** 2026-02-02

**Authors:** Patrick Yao, Ryan G Aronin, Clifford Pang, Akshay Syal, Yatindra Patel

**Affiliations:** 1 Department of Medicine, University of California Los Angeles David Geffen School of Medicine, Los Angeles, USA

**Keywords:** acute appendicitis, acute scrotum, scrotal abscess, scrotal pyocele, scrotal swelling

## Abstract

Laparoscopic appendectomy is generally considered safe with low overall morbidity, and scrotal complications are exceedingly rare. We report the case of a 65-year-old male who developed a large, complex right hydrocele complicated by abscess formation following laparoscopic appendectomy for acute appendicitis. This case highlights an unusual postoperative complication in an elderly adult patient without appendiceal perforation and emphasizes the importance of early recognition, multidisciplinary management, and consideration of this complication in the differential diagnosis of postoperative scrotal pain and swelling.

## Introduction

Laparoscopic appendectomy is one of the most common emergency general surgical procedures, with low overall morbidity [1,2]. While the procedure is associated with low overall morbidity, postoperative complications such as wound infection, intra-abdominal abscess, and ileus are well-documented [1,2]. However, extra-abdominal complications involving the inguinoscrotal region are exceedingly rare, particularly in the adult population.

Scrotal complications, including hydrocele, pyocele, or scrotal abscess, typically arise due to the persistence of a patent processus vaginalis (PPV). While the processus vaginalis obliterates in the majority of individuals during early childhood, autopsy and laparoscopic studies suggest it remains patent in approximately 12% of adults, serving as a potential conduit for peritoneal fluid and infection to track into the scrotum [3,4]. In the context of appendicitis, scrotal abscesses are almost exclusively associated with perforated appendicitis or generalized peritonitis [5-9]. We present a rare case of a 65-year-old male who developed a large complex hydrocele progressing to a scrotal abscess following laparoscopic appendectomy for non-perforated appendicitis. This case underscores the diagnostic challenge of postoperative scrotal swelling and highlights the potential for bacterial translocation even in the absence of gross appendiceal perforation.

## Case presentation

**Figure 1:**
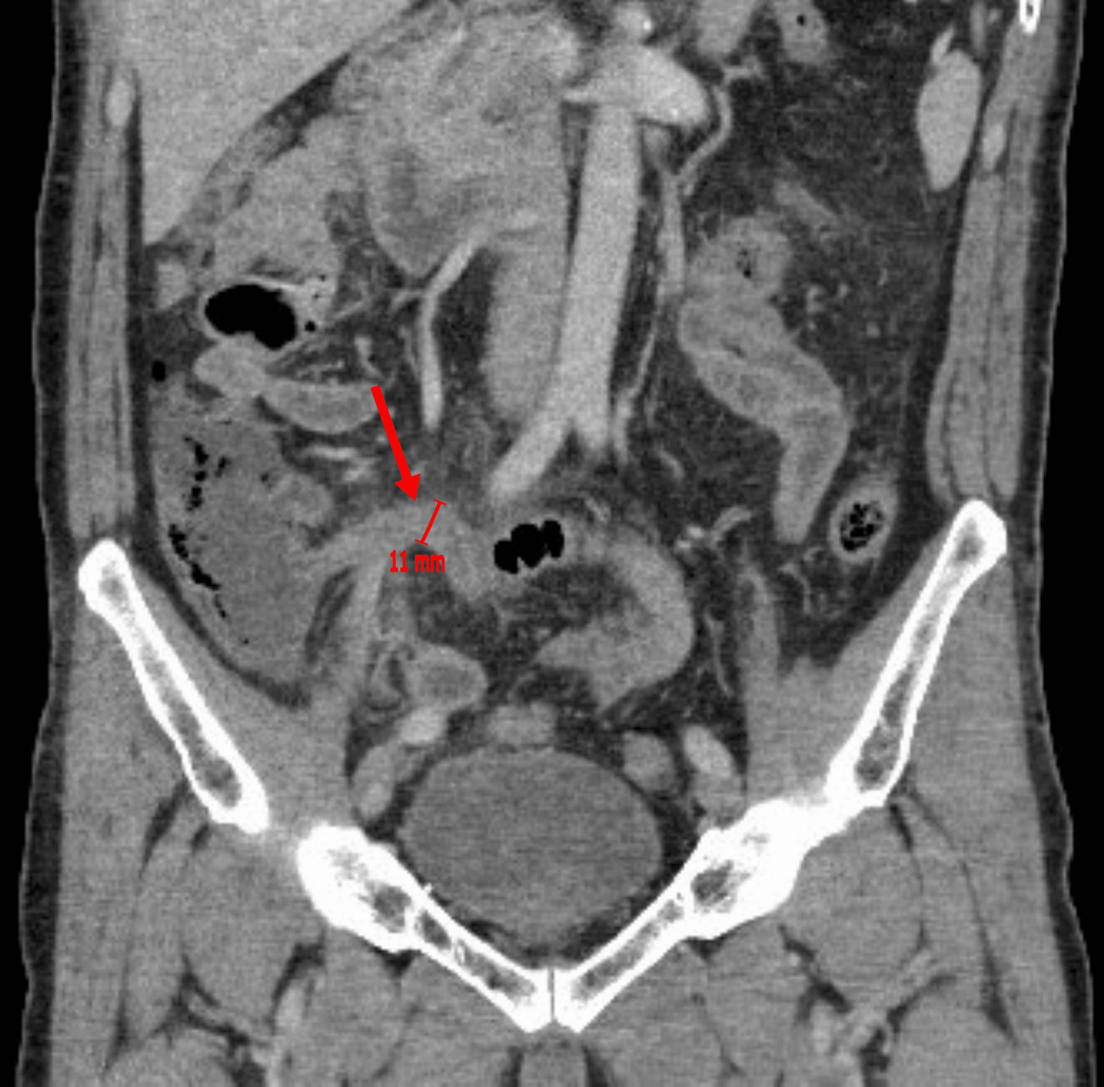
The preoperative CT abdomen/pelvis shows findings consistent with acute appendicitis without radiological evidence of perforation, revealing a dilated appendix (11 mm) labeled with an arrow.

A 65-year-old male with past medical history significant for Parkinson’s disease, hypertension, and nephrolithiasis presented with acute-onset right lower quadrant abdominal pain, associated with weakness, fever (101°F), nausea, and vomiting. He denied diarrhea, dysuria, or hematuria. On examination, he had focal right lower quadrant tenderness and mild generalized weakness. Initial labs showed elevated lactate of 3.6 mmol/L. CT abdomen/pelvis with contrast revealed a dilated appendix (11 mm) with abnormal enhancement, wall thickening, and adjacent fat stranding and edema consistent with acute appendicitis (Figure 1). He received intravenous fluids, ceftriaxone, and metronidazole, with lactate improving to 1.7 mmol/L. He subsequently underwent laparoscopic appendectomy without intraoperative complications. Postoperatively, he tolerated oral intake, had good pain control, and was discharged home the following day with oral amoxicillin/clavulanate potassium and analgesics.

**Figure 2:**
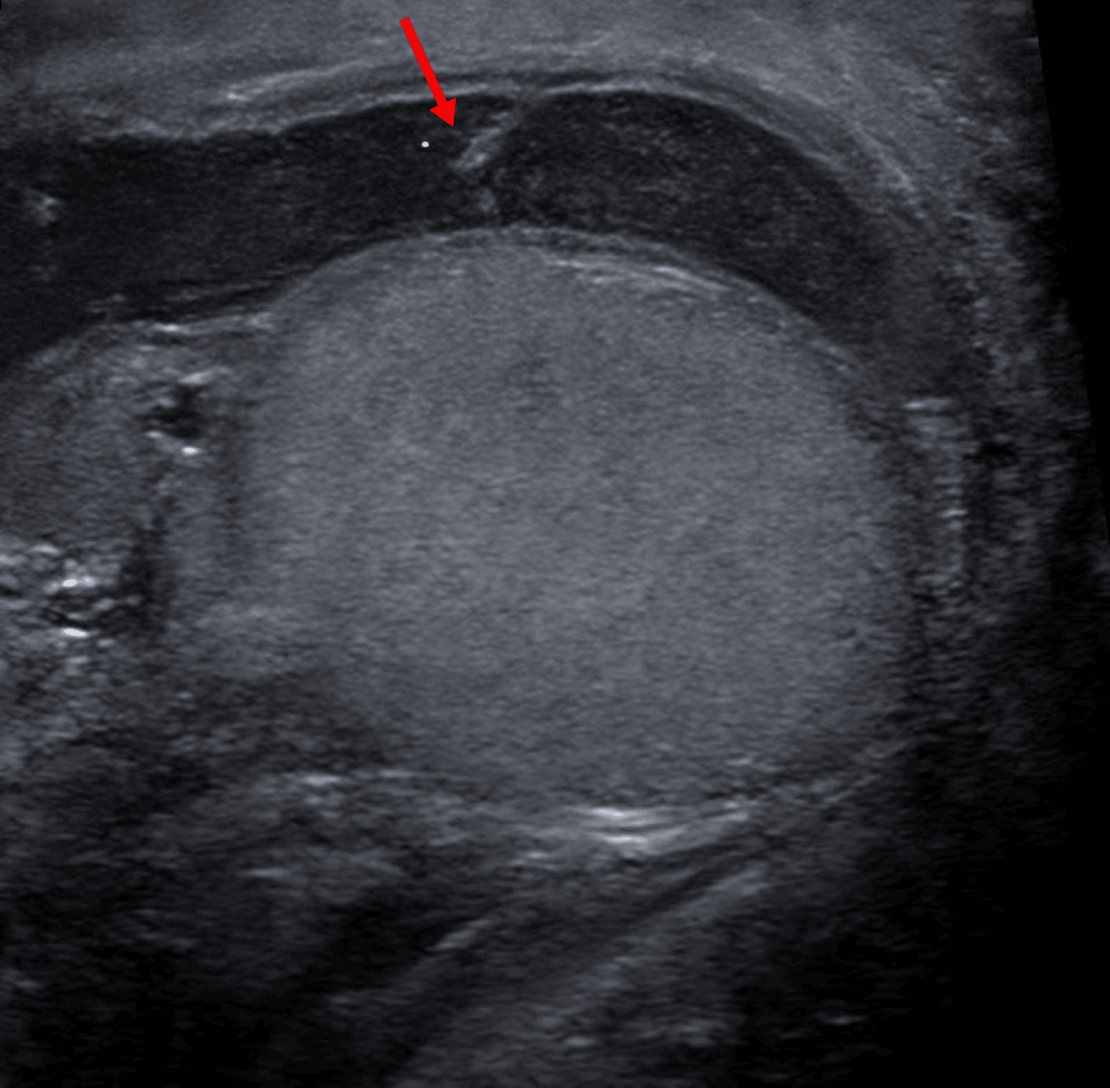
The ultrasound scan shows a large complex right hydrocele with internal septations and echoes labeled with an arrow.

Four days after surgery, the patient reached out to his surgeon with recurrent fever, progressive right scrotal pain, swelling, and difficulty ambulating. He was instructed to present to the emergency department (ED), where the patient was noted to have scrotal swelling and induration. Ultrasound of the scrotum revealed a large complex right scrotal fluid collection measuring 7.6 × 5.1 × 3.7 cm with internal septations and echoes, suggesting a complex hydrocele (Figure 2).

**Figure 3:**
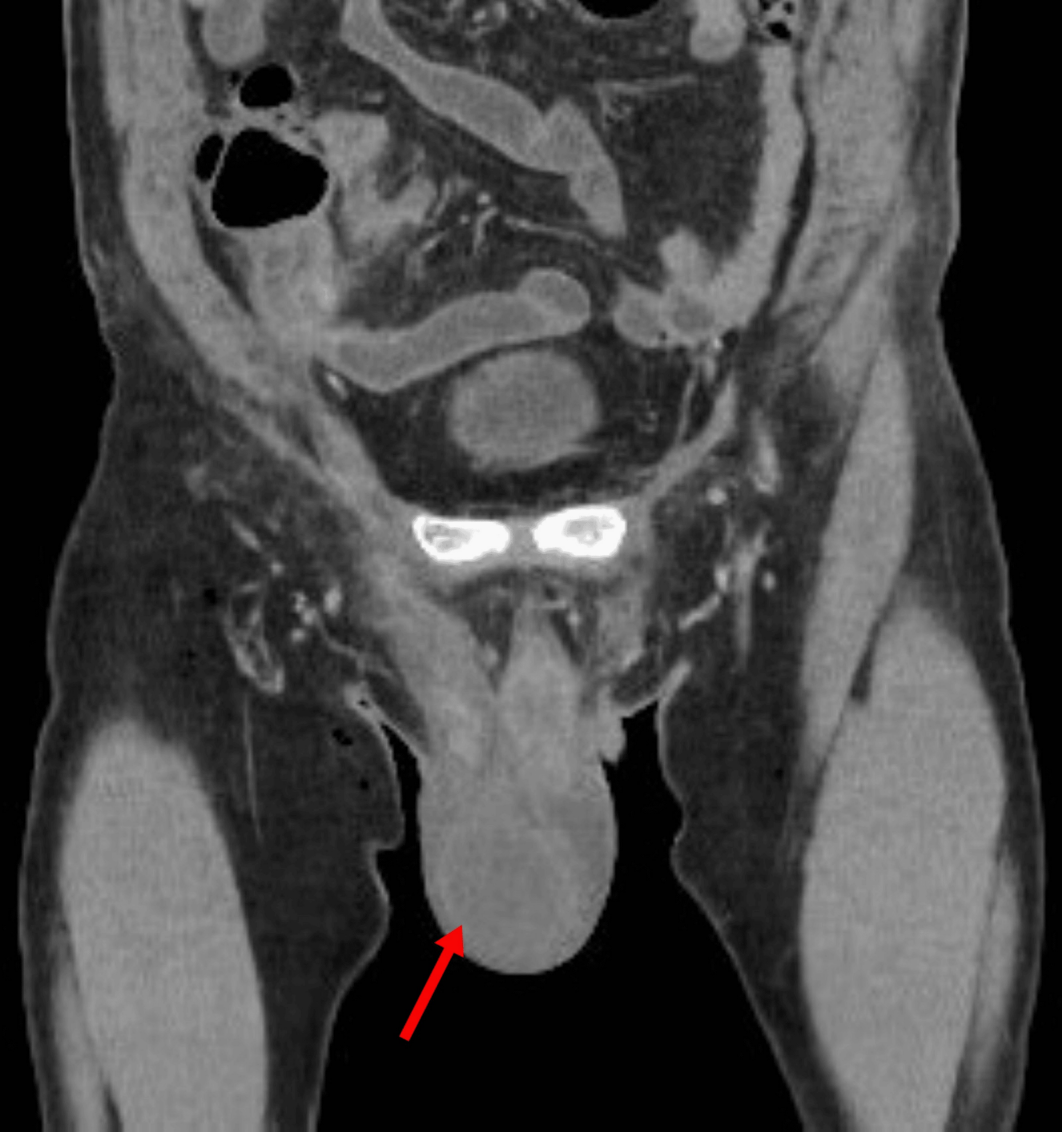
The CT scan shows findings of large right hydrocele labeled with an arrow.

**Figure 4:**
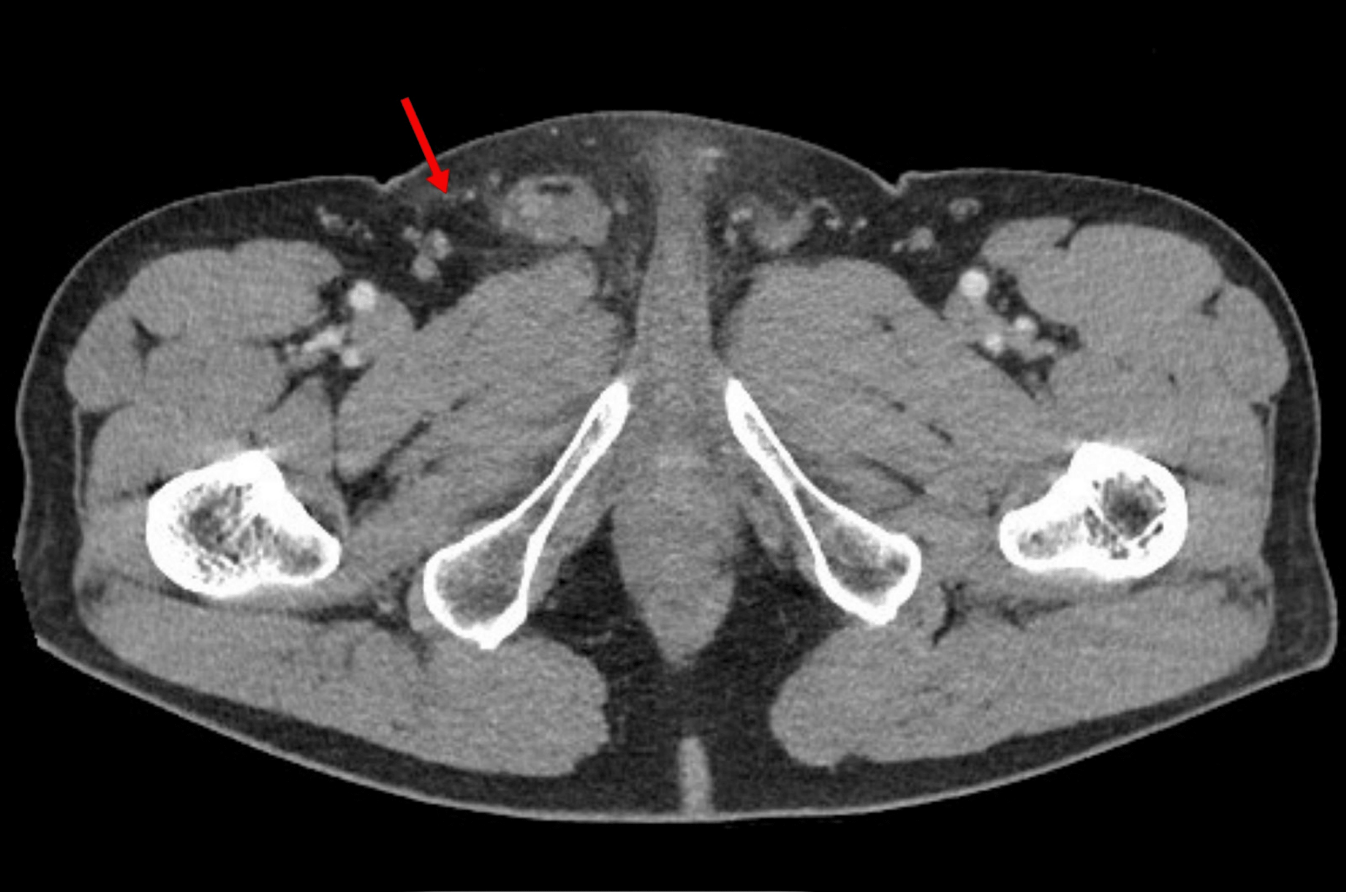
The CT scan shows lower abdominal wall inflammation, as evidenced by fat stranding labeled with an arrow.

The CT of abdomen and pelvis confirmed scrotal edema, large right hydrocele, and lower abdominal wall fat stranding (Figures 3, 4).

He was admitted for intravenous antibiotics with vancomycin and piperacillin-tazobactam. Infectious disease and urology were consulted. Blood cultures grew *Klebsiella pneumoniae*, confirming bacteremia. Conservative management with scrotal elevation, pain control, and close monitoring was recommended. The patient improved clinically and was discharged on oral amoxicillin/clavulanate potassium with outpatient urology follow-up.

At the outpatient follow-up six days post-discharge, the urologist noted a tense complex hydrocele and recommended hydrocelectomy once inflammation resolved. The patient later sought a second surgical opinion two days later due to persistent symptoms, including nausea, vomiting, and poor oral tolerance. The urologist speculated that the hydrocele may have resulted from a PPV allowing peritoneal fluid to track into the scrotum, but deferred drainage.

**Figure 5:**
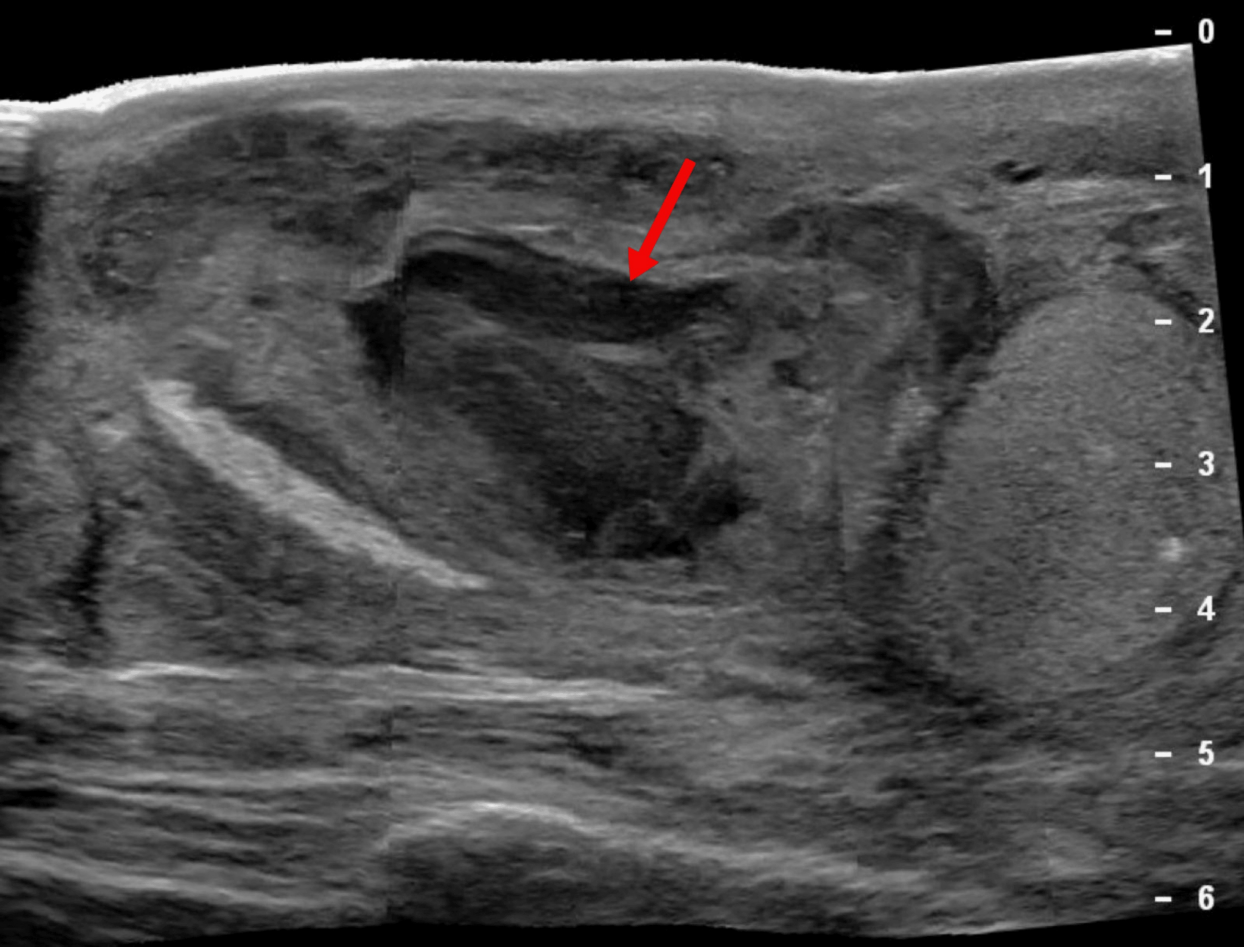
The ultrasound scan reveals findings concerning for a scrotal abscess, showing a right thick-walled extravesicular fluid collection superior to the right testis (arrow).

Nearly a month after his initial surgery, he presented again to the ED with worsening pain and purulent drainage from the right scrotum. Examination revealed a fluctuant scrotal mass with overlying erythema. Ultrasound of the scrotum showed a thick-walled, turbid fluid collection measuring 3.8 x 3.2 x 1.9 cm, suspicious for an abscess superior to the right testis (Figure 5).

Bedside incision and drainage (I&D) were performed, yielding purulent material. Cultures grew *Citrobacter freundii *complex, *Klebsiella pneumoniae*, and *Staphylococcus aureus*. He was initially treated with IV cefepime and daptomycin, transitioned to oral levofloxacin and cephalexin for two weeks, and discharged home with instructions for wound care and urology follow-up. It has been over one year since the initial surgery, and the patient has made a full recovery without any further complications (Table 1).

**Table 1:** Timeline of clinical events RLQ: Right lower quadrant.

Timeframe	Clinical Status	Investigation	Intervention/Management
Day 0	Presentation of RLQ abdominal pain	CT abdomen: acute appendicitis without perforation	Laparoscopic appendectomy
Day 1	Uncomplicated recovery	None	Discharged on oral antibiotics (amoxicillin/clavulanate)
Day 4	New scrotal pain/fever	Scrotal ultrasound: complex hydrocele; CT abdomen/pelvis: lower abdominal wall fat stranding and hydrocele; blood cultures: *Klebsiella pneumoniae*	Readmission; IV antibiotics (vancomycin and piperacillin-tazobactam)
Day 10	Clinical improvement	Physical exam: tense hydrocele	Discharged on oral antibiotics (amoxicillin/clavulanate)
Day 16	Persistent symptoms	Surgical second opinion	Continued observation; recommended hydrocelectomy once inflammation resolved; drainage deferred
Day 28	Purulent drainage; fluctuant mass	Repeat ultrasound: abscess; wound cultures: *Citrobacter freundii*, *Klebsiella pneumoniae*, and *Staphylococcus aureus*.	Readmission; bedside incision and drainage (I&D); IV antibiotics (cefepime and daptomycin)
Day 32	Clinical improvement	None	Discharged on oral antibiotics (levofloxacin and cephalexin) for 2 weeks
1 year	Asymptomatic	None	Complete recovery

## Discussion

Scrotal complications following laparoscopic appendectomy are an unusual clinical entity, with the vast majority of literature describing cases in the pediatric or adult population with perforated appendicitis [5-9]. The development of a scrotal abscess requires a pathway for infectious material to descend from the peritoneal cavity, most commonly in the form of a PPV. In adults, the incidence of PPV is often underestimated. Lee et al. and van Veen et al. both noted that PPV exists in roughly 12% of adult males undergoing unrelated laparoscopic procedures [3,4].

This case is unique for a couple of reasons. First, while Al Bazroon et al. reported the first middle-aged adult case of scrotal pyocele following perforated appendicitis in a 50-year-old male [7], our patient is of older, senior age, expanding the age range of adult patients affected by this rare complication. Second, this complication occurred despite the absence of appendiceal perforation or generalized peritonitis during the initial surgery. This phenomenon may be explained by the pneumoperitoneum created during laparoscopy, which increases intra-abdominal pressure and can force peritoneal fluid containing bacteria through a PPV that might otherwise have remained collapsed [10]. Furthermore, *K. pneumoniae*, one of the pathogens isolated in this case, is known for its high translocation potential [11]. It has been shown to invade and cross the intestinal epithelium via a transcellular pathway even in the absence of gross mucosal disruption [12].

Clinically, differentiating between a reactive hydrocele and a developing pyocele is challenging but critical. In this patient, initial imaging suggested a complex hydrocele [13]. The absence of frank purulence on imaging, lack of fluctuance on examination, and hemodynamic stability justified a trial of conservative management with broad-spectrum intravenous antibiotics and close monitoring. While the patient's early clinical improvement appeared to validate this approach, the eventual delay of nearly one month in diagnosis illustrates the deceptive nature of this complication. The antibiotic treatment for bacteremia likely suppressed the acute inflammatory response, leading to a subacute presentation that masked the severity of the infection.

Given these diagnostic difficulties, management of post-appendectomy scrotal swelling requires a high index of suspicion. While simple reactive hydroceles are typically managed conservatively with scrotal support, the presence of a loculated fluid collection in a septic patient warrants early surgical intervention. Delayed recognition of scrotal infections can lead to devastating complications, including testicular loss requiring orchiectomy [14] or progression to necrotizing fasciitis (Fournier’s gangrene) [15]. Therefore, in adult males presenting with acute scrotum following abdominal surgery, the threshold for urologic consultation and repeat imaging should be low.

## Conclusions

Post-appendectomy scrotal complications are exceedingly rare in adults but can be serious. Clinicians should maintain a high index of suspicion for infectious processes in adult patients presenting with a new onset of acute scrotum after appendectomy, particularly in the presence of a PPV. More importantly, even lesions that appear benign or noninfectious at the time of presentation, such as a postoperative hydrocele, may mask an evolving pyocele, which highlights the need for continued vigilance. Early imaging, prompt intervention, close follow-up, and multidisciplinary management are essential to prevent morbidity.
